# Upper respiratory tract CD8^+^ tissue-resident memory T cells protect against viral transmission

**DOI:** 10.1084/jem.20252329

**Published:** 2026-09-10

**Authors:** Sarah E. Michalets, Yixel Soto-Vazquez, Kathryn M. Moore, M. Elliott Williams, Ananya Saha, Ariana Jimenez, Yu-shan Huang, Anice C. Lowen, Christopher D. Scharer, Alison Swaims-Kohlmeier, Rustom Antia, Jacob E. Kohlmeier

**Affiliations:** 1 Department of Microbiology & Immunology, Emory University School of Medicine, Atlanta, GA, USA; 2 Department of Biology, Emory University, Atlanta, GA, USA; 3 Department of Gynecology & Obstetrics, Emory University School of Medicine, Atlanta, GA, USA

## Abstract

Intranasal vaccination elicits CD8 tissue-resident memory T cells (TRM) throughout the respiratory tract that provide cross-protection against heterosubtypic viral strains and reduce immunopathology. We previously demonstrated that CD8 TRM can reduce the probability of respiratory virus transmission and the magnitude of infection, using a murine model of parainfluenza virus transmission. Here, we show that CD8 TRM-mediated protection occurs independently of circulating leukocytes, B cells, or CD4 T cells. Additionally, we investigate the contributions of CD8 TRM in different respiratory tract compartments and illustrate that CD8 TRM in the upper respiratory tract (URT), but not the lower respiratory tract (LRT), become activated and proliferate in response to transmitted virus. Furthermore, we demonstrate that CD8 TRM in the URT alone provide sufficient immune surveillance to prevent propagation of infection after viral transmission. These findings offer insights into the development of T cell–based respiratory virus vaccines and shed light on the critical role of URT CD8 TRM in the prevention of viral transmission.

## Teaser

Michalets et al. demonstrate that upper respiratory tract tissue-resident memory CD8^+^ T cells are sufficient to protect against respiratory virus transmission, even in the absence of CD4 T cells, B cells, or lower respiratory tract immune responses, establishing a critical role for nasal CD8^+^ T cell surveillance in limiting respiratory virus spread.

## Introduction

While the ideal endpoint of vaccination is the induction of neutralizing antibodies and sterilizing immunity, respiratory virus infections such as SARS-CoV-2 and influenza have demonstrated the inadequacy of antibody-mediated immunity to provide broad protection against emerging and drifted viral variants (Cox et al., 2023; Doud et al., 2017; Greaney et al., 2021; Smith et al., 2004). In contrast, vaccine-induced T cell responses target conserved internal viral epitopes, enabling cross-protection among viral strains (Koutsakos et al., 2019; Meyer et al., 2023). As such, T cell–based vaccines represent a promising complement to conventional respiratory virus vaccines and circumvent the need for frequently updated strain-specific vaccines.

Tissue-resident memory T cells (TRM) are an essential cellular population of a mucosal vaccine–induced T cell response. Following a vaccination or viral infection, antigen-specific TRM localize in nonlymphoid tissues where they remain poised to rapidly initiate effector mechanisms and proliferate upon recognition of their cognate antigen (Mattingly et al., 2025). Unique transcriptional programs alter TRM’s trafficking capabilities and promote expression of canonical identification markers CD103 and/or CD69, enabling TRM to establish tissue residency and delineating them from their central memory and effector memory T cell counterparts (Mueller et al., 2013; Zheng and Wakim, 2022). CD8 TRM have been shown to mediate protection against heterosubtypic influenza virus and SARS-CoV-2 infections by significantly decreasing viral loads, limiting immunopathology, and preventing viral spread to the lungs (Grau-Exposito et al., 2021; Pizzolla et al., 2017a; Wu et al., 2014; Zens et al., 2016). While informative, these prior studies used high-dose infection models that delivered antigen throughout the respiratory tract and did not recapitulate natural viral transmission dynamics. To address this gap in knowledge, we employed a previously developed transmission model using Sendai virus, a murine parainfluenza virus that transmits between mice placed in direct contact and in shared airspace (Burke et al., 2013).

The respiratory tract is divided into the upper respiratory tract (URT), consisting of the nasal cavity, pharynx, and larynx, and the lower respiratory tract (LRT), comprising by the trachea, airways, and lung (Mettelman et al., 2022). The properties of TRM in the lung have been well defined and studied in the context of respiratory virus infections. However, an unappreciated role for cellular immunity in the nasal cavity of the URT is emerging, as the SARS-CoV-2 pandemic highlighted the lack of knowledge regarding vaccine-induced immune responses at this site. The question remains as to whether immune surveillance is required throughout the entire respiratory tract to prevent viral transmission, or if one anatomical compartment plays an outsized role in this process.

Here, we used a Sendai virus model of respiratory virus transmission to evaluate the contributions of TRM in the URT and LRT in preventing infection from transmission. We show that virus-specific TRM in the URT, but not the LRT, become activated through their TCRs, proliferate, and exert antiviral transcriptional programs in response to transmitted virus. Additionally, we demonstrate that TRM in the URT alone are sufficient to protect against respiratory virus transmission. These findings provide new evidence for the importance of vaccine-induced memory T cell responses in the URT and will inform future vaccine design aimed at preventing respiratory virus spread in the population.

## Results and discussion

### CD8 TRM reduce the probability and magnitude of infection following transmission

Following i.n. immunization with engineered viruses that express the immunodominant H-2K^d^ CD8 T cell epitope (FAPGNYPAL) from the Sendai virus nucleoprotein (SenNP), mice generate Sendai virus–specific central, effector, and respiratory tract TRM. Using a luciferase-encoding Sendai virus (Sendai-Luc) to visualize viral spread using in vivo bioluminescence imaging, we previously demonstrated that Sendai-specific CD8 TRM could protect against developing a productive infection following transmission (Uddbäck et al., 2024).

**Figure 1.**
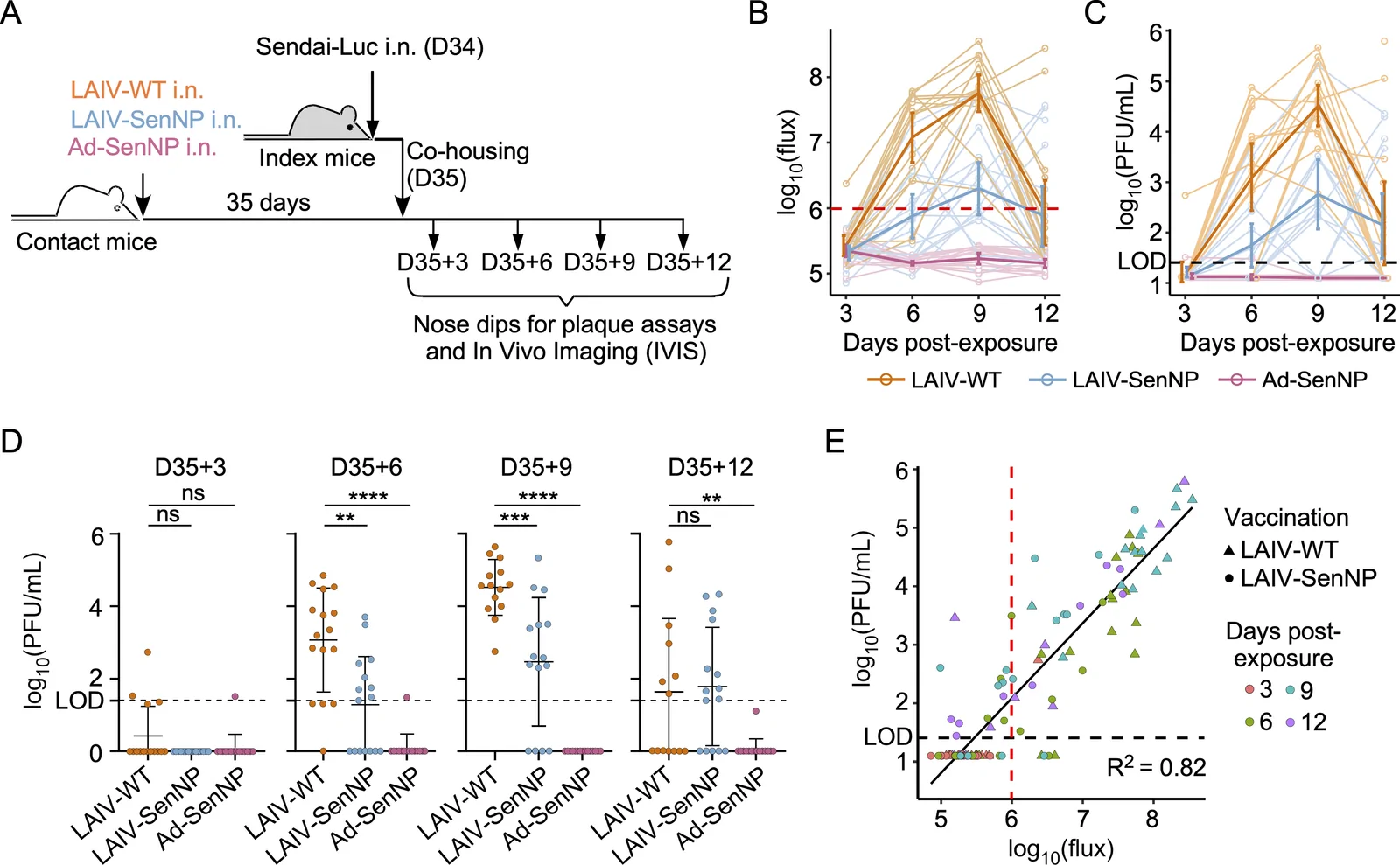
**Vaccine-induced CD8 TRM reduce the susceptibility and magnitude of infection following transmission. (A)** Experimental schematic where contact mice immunized i.n. with LAIV-WT, LAIV-SenNP, or Ad-SenNP were co-housed with a Sendai-Luc–infected index mouse, and transmission was assessed by in vivo imaging and nasal shedding titers. **(B)** Bioluminescence curves of LAIV-WT, LAIV-SenNP, and Ad-SenNP contact mice (*n* = 14–16 per group) following co-housing with index mice. **(C)** Nasal shedding kinetics curves of immunized contact mice. **(D)** Nasal shedding titers of immunized contact mice at 3, 6, 9, and 12 days after co-housing. **(E)** Correlation of nasal shedding titers with bioluminescent flux. For B and C, solid dark lines represent means, solid pale lines represent individual mice, dashed grey lines represent the limit of detection, and dashed red lines represent the threshold of infection. Error bars (B and C) represent 95% binomial confidence intervals or standard deviation (D). All data are combined from at least two independent replicates. Statistical significance was determined using a two-sided Mann–Whitney test with **P < 0.01, ***P < 0.001, ****P < 0.0001, and NS for not significant.

To extend these findings, we first compared in vivo bioluminescent imaging results with nasal shedding of infectious virus. Contact mice were immunized i.n. with a live-attenuated PR8 influenza A virus encoding the FAPGNYPAL epitope for SenNP (LAIV-SenNP) or a replication-deficient adenoviral serotype 5 vector expressing the SenNP (Ad-SenNP). After 35 days, mice were co-housed with a Sendai-Luc–infected index mouse. Transmission from the infected index mouse to the immunized contact mice was tracked longitudinally by nose dips for viral shedding analysis and in vivo imaging (Fig. 1 A). A control group was inoculated with a live-attenuated influenza A virus lacking the SenNP antigen (LAIV-WT) to account for the role of inflammation or trained innate immunity during immunization, without generating any Sendai-specific adaptive immunity. Infection was evident by both bioluminescent flux and viral titers in LAIV-WT and LAIV-SenNP–immunized mice by 6 days after co-housing (D35 + 6), and the LAIV-SenNP group exhibited significantly reduced nasal shedding at D35 + 6 and D35 + 9. Notably, mice immunized with Ad-SenNP did not exhibit nasal shedding at any time point (Fig. 1, B and D). Differences between LAIV-SenNP and Ad-SenNP–immunized mice can be attributed to the number of Sendai-specific TRM elicited during vaccination, as we previously showed that LAIV-SenNP elicits fewer CD8 TRM in the respiratory tract, resulting in decreased protection compared with Ad-SenNP (Uddbäck et al., 2024). Nasal shedding titers correlated strongly with bioluminescent flux in the respiratory tract for all time points evaluated (Fig. 1 E). While these findings demonstrate that CD8 TRM can significantly limit both viral propagation and nasal shedding following transmission, they do not exclude potential contributions from other immune cell populations.

**Figure 2.**
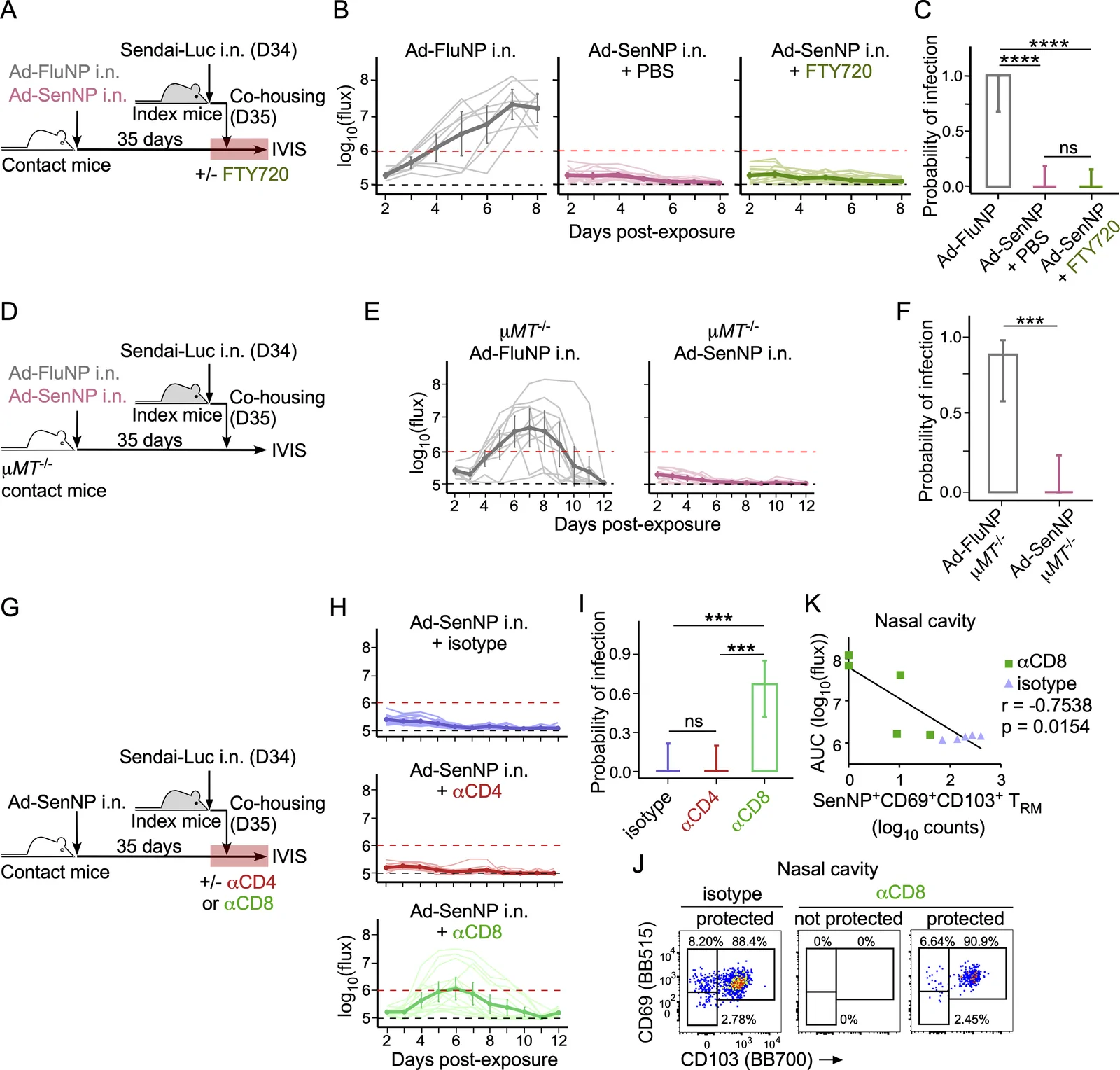
**Circulating immune cells, B cells, and CD4 T cells are not necessary to prevent infection from transmission when CD8 TRM are present. (A)** Experimental schematic where Ad-FluNP i.n. or Ad-SenNP i.n. immunized contact mice were co-housed with Sendai-Luc–infected index mouse and treated with FTY720 or PBS control i.p. **(B)** Bioluminescence curves of Ad-FluNP i.n. (*n* = 8), Ad-SenNP i.n. with PBS (*n* = 16), and Ad-SenNP i.n. with FTY720 (*n* = 20) contact mice following co-housing with index mice. **(C)** Probability of infection for immunized contact mice, calculated as the proportion of contact mice that became infected. **(D)** Experimental schematic where μMT^−/−^ mice were immunized i.n. with Ad-FluNP or Ad-SenNP prior to co-housing with Sendai-Luc–infected index mouse. **(E)** Bioluminescence curves of μMT^−/−^ mice immunized with Ad-FluNP (*n* = 10) and Ad-SenNP (*n* = 12). **(F)** Probability of infection for μMT^−/−^ contact mice immunized with Ad-FluNP and Ad-SenNP. **(G)** Experimental schematic to assess the role of CD4 and CD8 T cells where Ad-SenNP i.n. immunized contact mice were administered anti-CD4 or anti-CD8–depleting monoclonal antibody every 3 days, starting 1 day prior to co-housing with a Sendai–Luc–infected index mouse. **(H)** Bioluminescence curves of contact mice (*n* = 16/group) immunized with Ad-SenNP and treated with isotype control, anti-CD4, or anti-CD8 antibodies. **(I)** Probability of infection of immunized contact mice. **(J)** Example flow cytometry plots of isotype- and anti-CD8–treated mice at the conclusion of co-housing. Plots show CD69 and CD103 expression gated on IV^−^ SenNP^+^ CD8^+^ T cells in the nasal cavity. **(K)** Correlation between the AUC of the bioluminescence signal from Sendai-Luc infection and the number of SenNP^+^ CD69^+^ CD103^+^ remaining in the nasal cavity after anti-CD8 depletion. For B, E, and H, solid dark lines represent means, solid pale lines represent individual mice, dashed grey lines represent the limit of detection, and dashed red lines represent the threshold of infection. Error bars (B–I) represent 95% binomial confidence intervals. Data are combined from at least two independent replicates. Statistical significance was determined using a two-sided Mann–Whitney test (C, F, and I) and nonparametric Spearman correlation (K), with ***P < 0.001, ****P < 0.0001, and NS for not significant.

**Figure S1.**
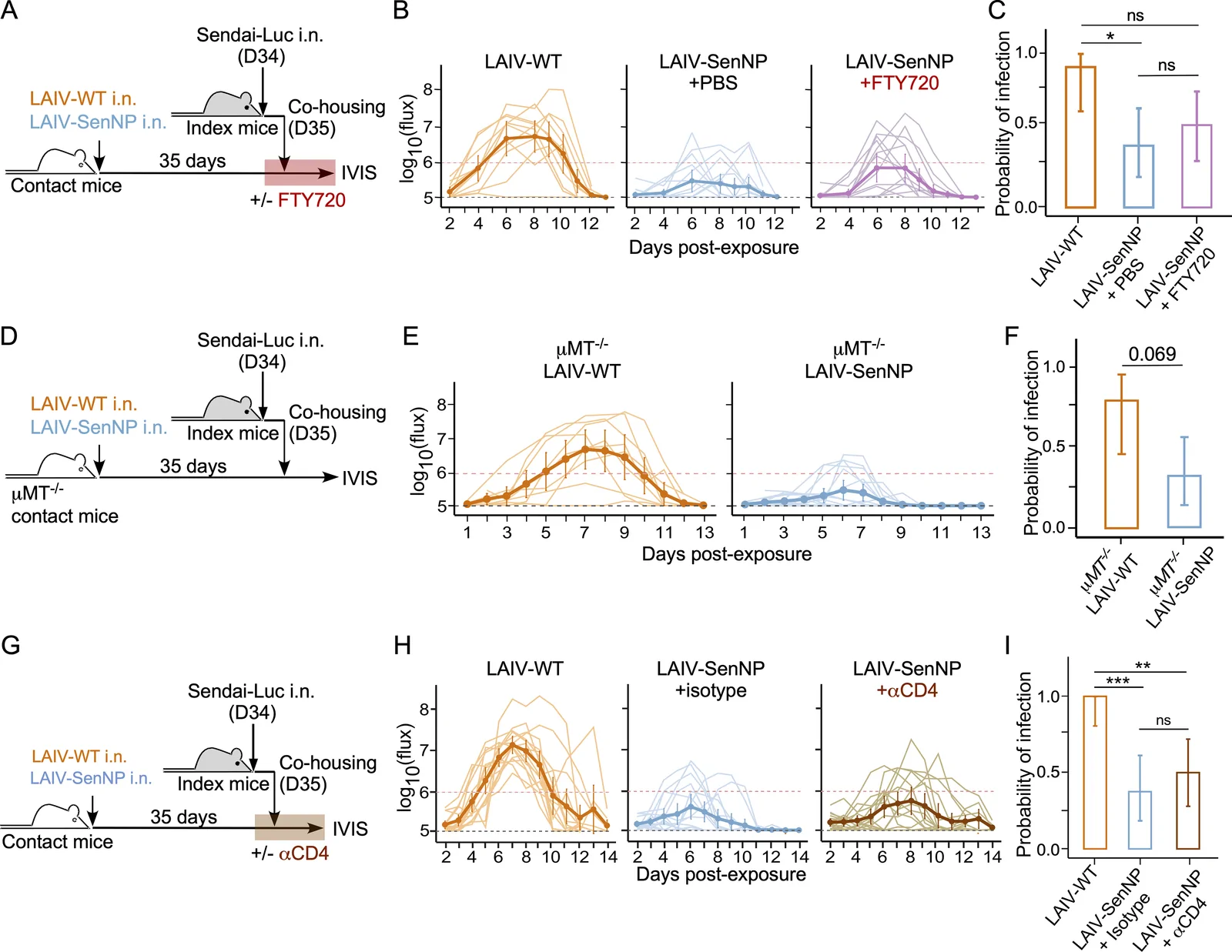
**Circulating effector cells, B cells, and CD4 T cells are dispensable for protection against transmission even when a lower quantity of respiratory tract TRM are present. (A)** Experimental schematic where LAIV-WT i.n. or LAIV-SenNP i.n. immunized contact mice were co-housed with Sendai-Luc–infected index mice and treated with FTY720 or PBS control i.p. **(B)** Bioluminescence curves of LAIV-WT i.n. (*n* = 11), LAIV-SenNP i.n. with PBS (*n* = 15), and LAIV-SenNP i.n. with FTY720 (*n* = 15) contact mice following co-housing with index mice. **(C)** Probability of infection for immunized contact mice, calculated as the proportion of contact mice that became infected. **(D)** Experimental schematic where μMT^−/−^ mice were immunized i.n. with LAIV-WT or LAIV-SenNP prior to co-housing with Sendai-Luc–infected index mouse. **(E)** Bioluminescence curves of μMT^−/−^ mice immunized with LAIV-WT (*n* = 9) and LAIV-SenNP (*n* = 16). **(F)** Probability of infection for μMT^−/−^ contact mice immunized with LAIV-WT and LAIV-SenNP. **(G)** Experimental schematic where LAIV-WT i.n. or LAIV-SenNP i.n. immunized contact mice were treated with isotype or anti-CD4–depleting antibodies and co-housed with Sendai-Luc–infected index mice. **(H)** Bioluminescence curves of mice immunized with LAIV-WT (*n* = 16), LAIV-SenNP and treated with isotype antibody (*n* = 16), and LAIV-SenNP and treated with anti-CD4 antibody (*n* = 16). **(I)** Probability of infection for isotype- and anti-CD4–treated mice immunized with LAIV-WT or LAIV-SenNP. For B, E, and H, solid dark lines represent means, solid pale lines represent individual mice, dashed grey lines represent the limit of detection, and dashed red lines represent the threshold of infection. Error bars (C, F, and I) represent 95% binomial confidence intervals. All data are combined from two independent replicates. Statistical significance was determined using a two-sided Mann–Whitney test with *P < 0.05, **P < 0.01, ***P < 0.001, and NS for not significant.

**Figure S2.**
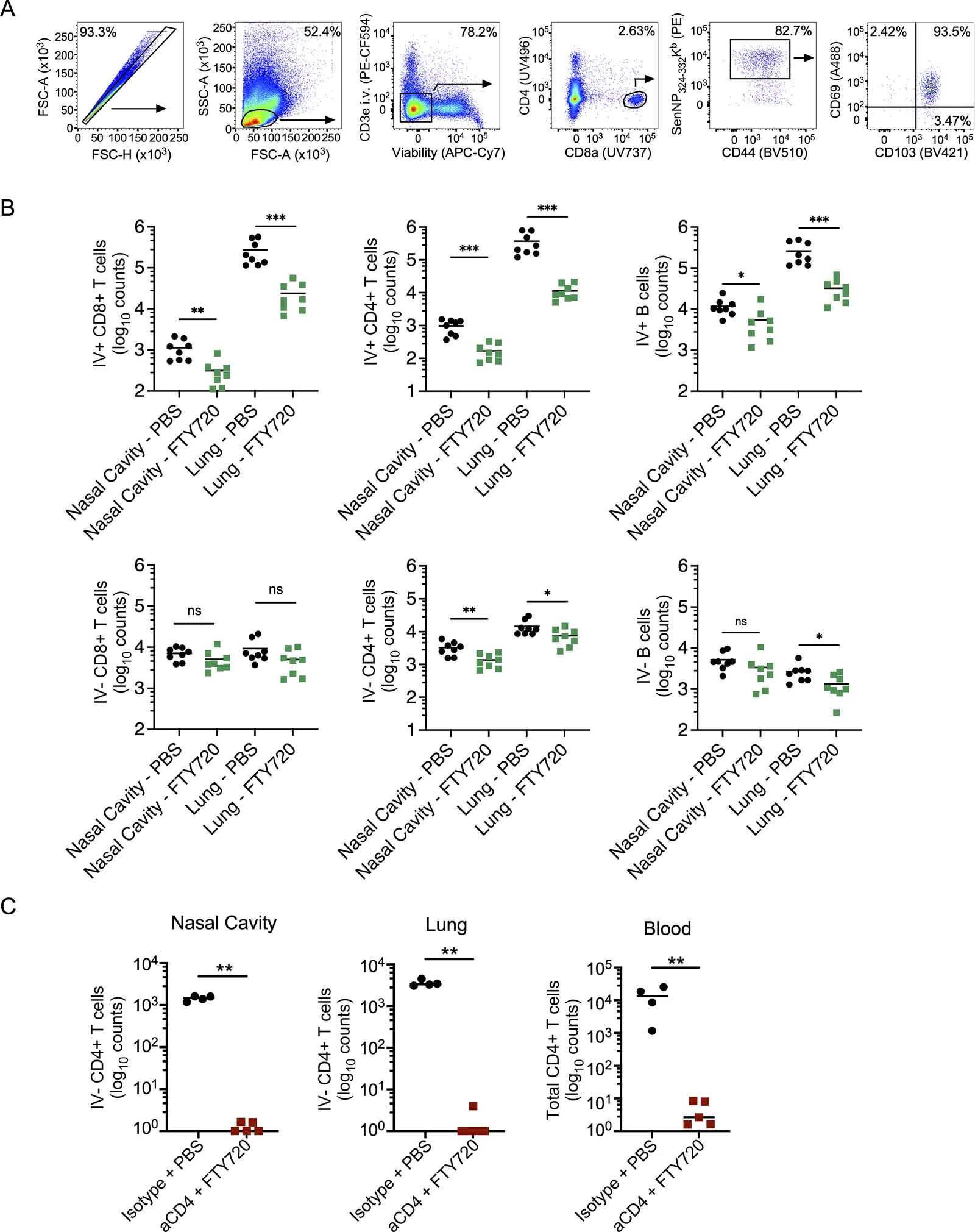
**Flow cytometry gating strategy and confirmation of FTY720 and anti-CD4 depletion efficacy. (A)** Gating strategy for identification of IV^−^ CD8^+^ SenNP^+^ CD69^+^ CD103^+^ T cells in Figs. 2, 3, 4, and 5. **(B)** The number of IV^+^ (top row) and IV^−^ (bottom row) total CD8 T cells (left column), CD4 T cells (middle column), and B cells (right column) in the nasal cavity and lung of vehicle (PBS) (*n* = 8) or FTY720-treated Ad-SenNP-immunized mice (*n* = 8). **(C)** The number of IV^−^ CD4 T cells in the nasal cavity and lung, and total CD4 in the blood, of isotype + PBS (*n* = 4) and anti-CD4 + FTY720–treated mice (*n* = 5). Statistical significance was determined using a two-sided Mann–Whitney test with *P < 0.05, **P < 0.01, ***P < 0.001, and NS for not significant.

To further define the sufficiency of CD8 TRM in protection against respiratory virus transmission, we evaluated whether the influx of circulating immune cells, B cells, or CD4 T cells was required for protection in this model. We utilized FTY720 dosing to inhibit S1P-dependent cellular trafficking and evaluate whether circulating memory CD8 T cells and other S1P-dependent leukocytes aid in protection against transmission. Substantial decreases in circulating IV^+^, but not IV^−^, B cells, CD8^+^, and CD4^+^ T cells were confirmed in the nasal cavity and lung following FTY720 administration (Fig. S2 B). Contact mice were immunized i.n. with Ad-SenNP or an adenoviral vector control (Ad-FluNP), and in vivo imaging was performed to monitor transmission of Sendai-Luc from the index mouse to immunized contact mice (Fig. 2, A and B). As expected, all contact mice (eight out of eight) who received the Ad-FluNP control and lacked any Sendai-specific immunity became infected. Interestingly, regardless of whether S1P-dependent cellular trafficking was inhibited, all animals with SenNP-specific CD8 TRM were protected from infection (Fig. 2, B and C). To determine whether B cell responses elicited during immunization were required for protection against transmission, we used μMT^−/−^ mice which lack functional mature B cells. Unlike LAIV-SenNP, which only encodes the 9-amino acid FAPGNYPAL sequence from SenNP and is thus extremely unlikely to induce any Sendai-specific B cells, Ad-SenNP expresses the entire SenNP and could potentially lead to SenNP-specific B cell responses after immunization. We immunized μMT^−/−^ contact mice with Ad-SenNP or Ad-FluNP as a control and challenged them using the Sendai-Luc transmission model (Fig. 2 D). 90% (9 out of 10) of Ad-FluNP–immunized μMT^−/−^ contact mice became infected, while all (12 out of 12) of the Ad-SenNP–immunized μMT^−/−^ contact mice were protected from infection, mimicking the results observed in WT mice (Fig. 2, E and F). These results were reiterated using LAIV-SenNP immunization. Although increased breakthrough infections occurred following LAIV-SenNP priming due to the lower number of TRM induced compared with Ad-SenNP priming, there was no difference in the probability of infection between PBS control and FTY720-treated groups (Fig. S1, A–C), and μMT^−/−^ mice primed with LAIV-SenNP showed a similar trend in protection (Fig. S1, D–F).

CD4 T cells have been shown to provide “help” and amplify memory CD8 T cell responses through cytokine secretion and co-stimulation (Laidlaw et al., 2016; Novy et al., 2007). To evaluate whether bystander CD4 T cells were necessary for CD8 TRM-mediated protection, we vaccinated contact mice with Ad-SenNP and administered either an isotype control or CD4-depleting antibody during Sendai–Luc transmission (Fig. 2 G). As expected, all mice were protected against Sendai infection, regardless of whether CD4 T cells were depleted (Fig. 2, H and I). Similar results were obtained following LAIV-SenNP immunization (Fig. S1, G–I). In contrast, administration of a CD8-depleting antibody resulted in significant loss of protection (Fig. 2, H and I). We noted that the loss of protection following anti-CD8 depletion was not absolute (38%, 6 out of 16 mice remained protected) and further investigated these mice immediately following co-housing. We observed that the efficacy of SenNP-specific TRM depletion in the nasal cavity varied between mice, and that the number of nasal cavity SenNP-specific TRM remaining at the conclusion of co-housing significantly correlated with the viral load during transmission (Fig. 2, J and K; and gating: Fig. S2). Taken together, these findings show that CD8 TRM in isolation can protect against respiratory virus transmission, independent of circulating immune cells, B cell responses, or CD4 T cell help.

### Antigen-specific CD8 T cells in the URT respond to transmitted virus

Although i.n. vaccination to induce mucosal immune responses is highly appealing, there are concerns over the feasibility and safety of generating virus-specific memory T cells in the lungs of human recipients. Previous work has shown that antigen must be delivered locally to the lung to elicit TRM (McMaster et al., 2018), which may necessitate inhalation of large doses and cause adverse side effects in vaccine recipients. Due to these concerns, we sought to investigate whether the URT or LRT compartments required T cell surveillance to prevent infection following transmission.

**Figure 3.**
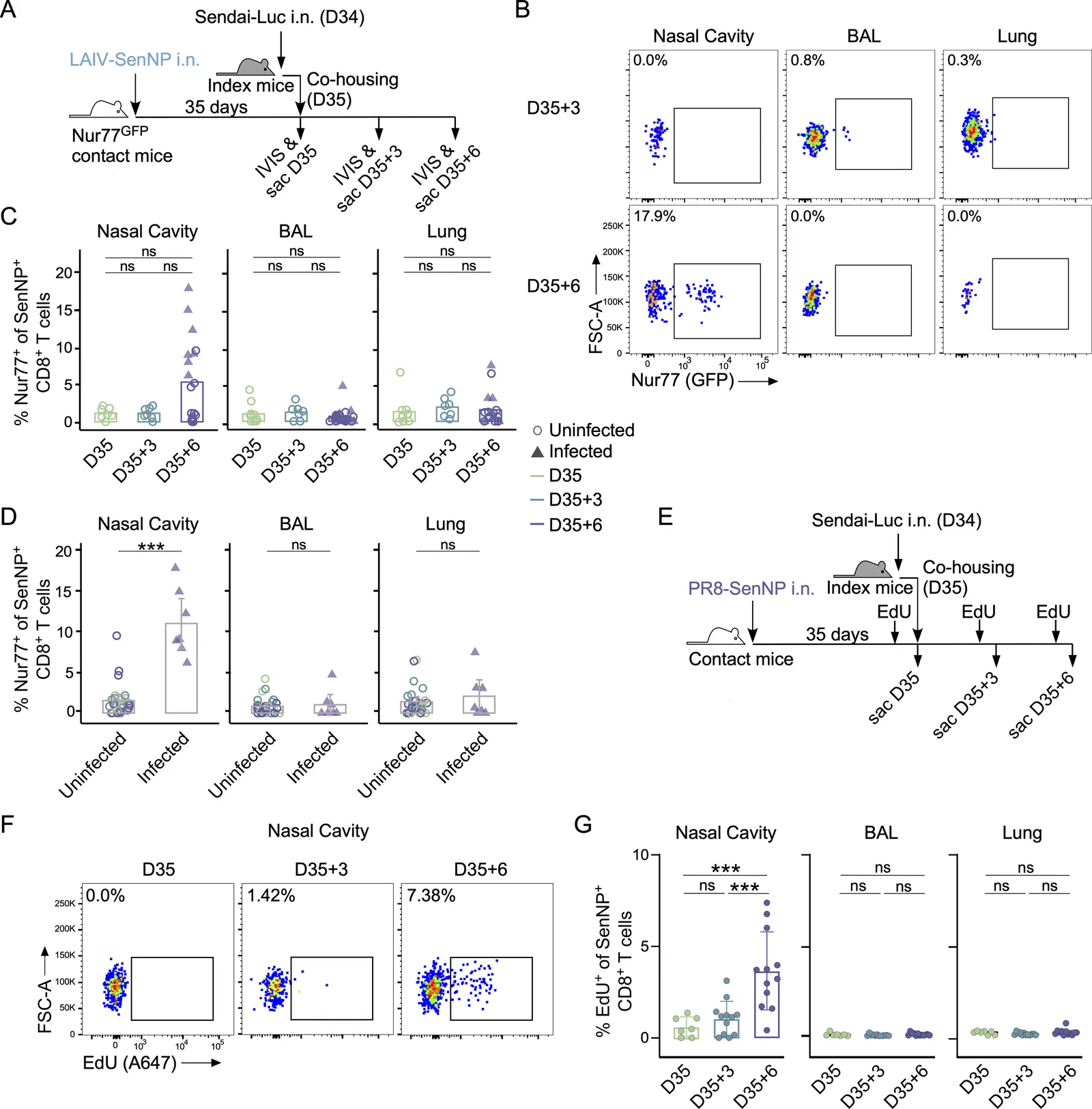
**Antigen-specific CD8 T cells in the URT, but not LRT, become activated and proliferate in response to transmitted virus. (A)** Experimental schematic in which *Nur77*^*GFP*^ contact mice immunized i.n. with LAIV-SenNP were co-housed with a Sendai-Luc–infected index mouse and sacrificed at D35 + 0 (*n* = 10), D35 + 3 (*n* = 7), or D35 + 6 (*n* = 19). **(B)** Representative flow cytometry plots of Nur77 expression gated on IV^−^ CD8^+^ SenNP^+^ T cells in immunized contact mice. **(C)** Frequency of Nur77^+^ IV^−^ CD8^+^ SenNP^+^ T cells in the nasal cavity, BAL, and lung of immunized mice at D35 + 0, D35 + 3, and D35 + 6. Circles represent uninfected mice, and triangles represent infected mice by in vivo imaging. **(D)** Frequency of Nur77+ of IV− CD8^+^ SenNP+ T cells separated by infection status. **(E)** Experimental schematic to assess T cell proliferation, where PR8-SenNP i.n. immunized contact mice were injected with 1 mg EdU i.p. 1 day prior to sacrifice at D35 + 0 (*n* = 7), D35 + 3 (*n* = 12), or D35 + 6 (*n* = 12) after co-housing with index mouse. **(F)** Representative flow cytometry plots of EdU expression gated on IV^−^ CD8^+^ SenNP^+^ T cells in immunized contact mice. **(G)** Frequency of EdU^+^ IV^−^ CD8^+^ SenNP^+^ T cells in the nasal cavity, BAL, and lung of immunized contact mice at D35 + 0, D35 + 3, and D35 + 6. Data are combined from three (B–D) or two (F and G) independent replicates. Statistical significance was determined using a two-sided Mann–Whitney test with ***P < 0.001, and NS for not significant.

We first examined the localization of virus-specific T cell activation during transmission. We used Nur77^GFP^ reporter mice as a readout for T cell activation, where TCR stimulation results in GFP expression (Moran et al., 2011). Nur77^GFP^ contact mice were immunized with LAIV-SenNP, co-housed with Sendai-Luc–infected index mice, and sacrificed at 3 days (D35 + 3) and 6 days (D35 + 6) after co-housing to assess GFP expression in SenNP-specific CD8 T cells in different areas of the respiratory tract (Fig. 3 A). GFP^+^ Sendai virus–specific T cells were observed in the nasal cavity at D35 + 6, whereas no T cell activation was seen in the airways (bronchoalveolar lavage, BAL) or lung at either time point (Fig. 3, B and C). Due to the large number of mice at D35 + 6 (12 out of 19) that were not infected, as assessed by in vivo imaging (represented by open circles), the frequency of GFP^+^ SenNP^+^ CD8 T cells at D35 + 6 in the nasal cavity showed a trending increase but did not reach statistical significance. When stratified according to infection status, we observed that infected animals had a significant enrichment of activated antigen-specific CD8 T cells in the URT, but not LRT (Fig. 3 D). We next assessed whether Sendai virus–specific CD8 T cells were proliferating in the URT or LRT in response to transmitted virus by 5-ethynyl-2′-deoxyuridine (EdU) labelling. WT mice were vaccinated, co-housed for Sendai-Luc transmission after 35 days, and dosed with EdU 24 h prior to sacrifice at D35, D35 + 3, or D35 + 6 (Fig. 3 E). Consistent with our previous results, EdU was detected in SenNP-specific CD8 T cells within the nasal cavity at D35 + 6, but not within the BAL or lungs at either time point (Fig. 3, F and G). These findings establish the nasal cavity, or URT, as the predominant site of the T cell response to transmitted respiratory virus and highlight the need to characterize the role of URT TRM in preventing respiratory virus transmission.

### Activated nasal cavity–resident memory CD8 T cells undergo antiviral effector transcriptional changes

**Figure 4.**
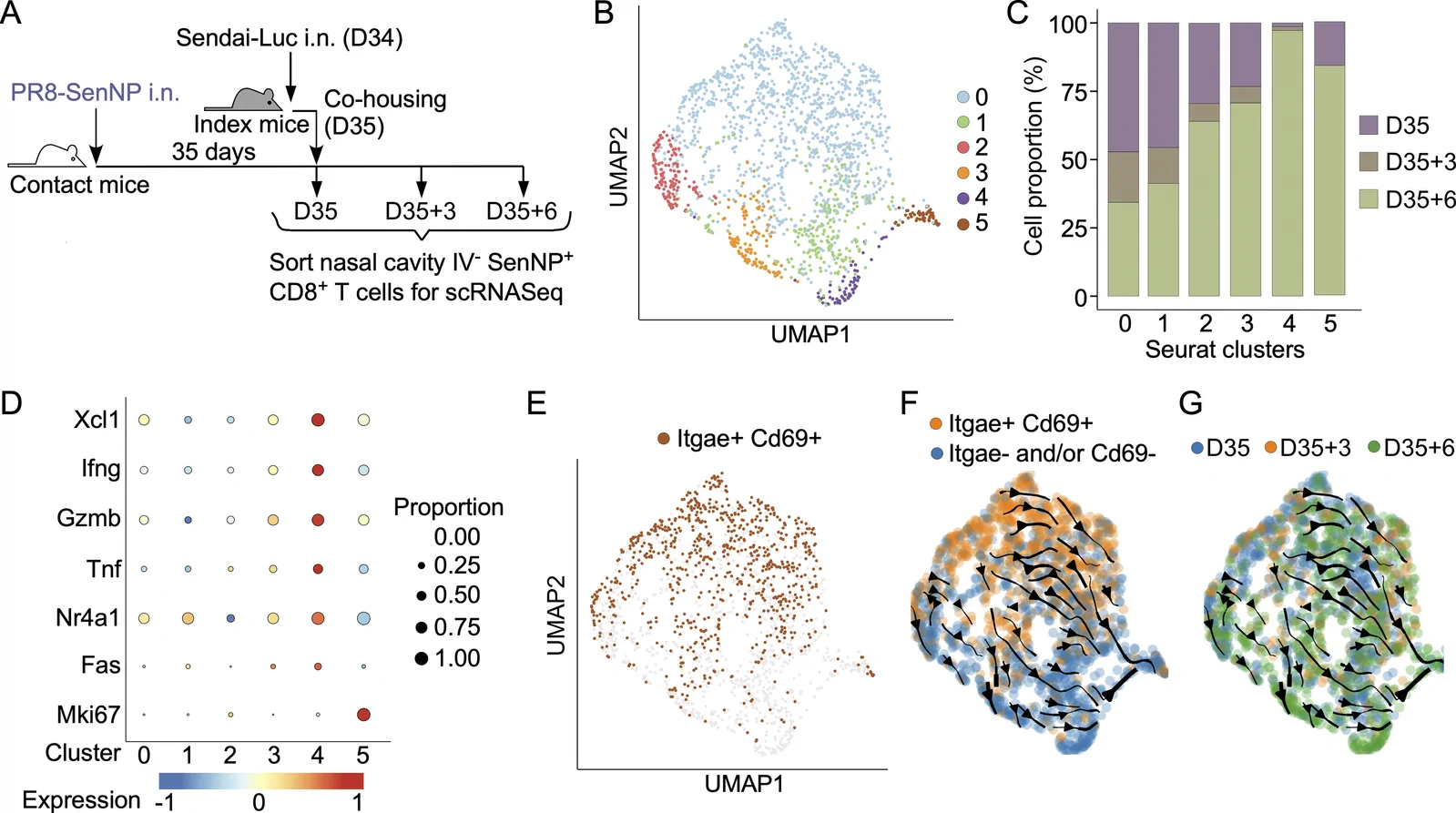
**URT-resident memory CD8 T cells initiate antiviral effector transcriptional programs during respiratory virus transmission. (A)** Experimental schematic where PR8-SenNP i.n. immunized contact mice were co-housed with a Sendai-Luc–infected index mouse, and SenNP^+^ CD8^+^ T cells from the nasal cavity were sorted for single-cell RNA sequencing at D35 + 0, D35 + 3, and D35 + 6. **(B)** UMAP projection of nasal cavity SenNP^+^ IV^−^ CD8^+^ T cells from contact mice during Sendai-Luc transmission. **(C)** Composition of UMAP Seurat clusters by day post co-housing. **(D)** Bubble plot with expression of effector function genes for each cluster. **(E)** Itgae^+^ Cd69^+^ cells overlaid on UMAP. **(F)** RNA velocity analysis with streamlines in the direction of cellular trajectory and Itgae and Cd69 expression status overlaid. **(G)** RNA velocity with time point post co-housing overlaid. Data are representative of two independent experimental replicates.

To further understand the activation kinetics and effector programs of URT resident memory CD8 T cells during viral transmission, we FACS-sorted intravital antibody-negative, SenNP^+^ CD8^+^ T cells from the nasal tissue of PR8-SenNP i.n. immunized contact mice after co-housing with a Sendai-Luc–infected index mouse and performed single-cell RNA sequencing (Fig. 4 A). Cells stratified into six clusters; clusters 4 and 5 predominantly consisted of cells from the D35 + 6 time point, while clusters 0 through 3 were a heterogeneous mix from all time points (Fig. 4, B and C). Cluster 4 exhibited increased expression of many canonical CD8 T cell effector function genes, including *Ifng*, *Gzmb*, and *Tnf* (Fig. 4 D). *Nr4a1* (Nur77) expression was upregulated in cluster 4, aligning with our previous results that Nur77^GFP^ expression denotes activated nasal cavity SenNP-specific CD8 T cells following transmission. Nearly all of the cells in cluster 5 expressed *Mki67*, indicating that this cluster represents proliferating SenNP^+^ CD8 T cells. Notably, cells in clusters 0 through 3 had low to non-detectable levels of expression for these activation genes, indicating that these cells likely represent SenNP-specific CD8 T cells that are present at steady state after priming but have not encountered their cognate antigen following transmission. It is important to note that we sorted all SenNP^+^ IV^−^ CD8^+^ T cells from the nasal cavities of these mice, a population which includes TRM T cells, effector memory T cells, and activated effector T cells. Cells that expressed canonical TRM marker genes *Cd69* and *Itgae*, which encodes CD103, were dispersed throughout clusters 0 through 3. Conversely, very few *Itgae*^+^*Cd69*^+^ cells were identified in the activated and proliferating cells of clusters 4 and 5 (Fig. 4 E). We hypothesized that these activated cells in clusters 4 and 5 likely originated from TRM and performed RNA velocity analysis to visualize the cellular transitions occurring between clusters. Streamlines from TRM-rich regions were directed toward the activated clusters, in accordance with the time points of sample collection and corroborating the notion that these activated effector cells arise from TRM (Fig. 4, F and G). These results support the nasal cavity as the critical site for TRM activation during transmission, as cells in this location rapidly transition to and adopt antiviral effector transcriptional states.

### URT CD8 TRM are sufficient to prevent infection from transmission

**Figure 5.**
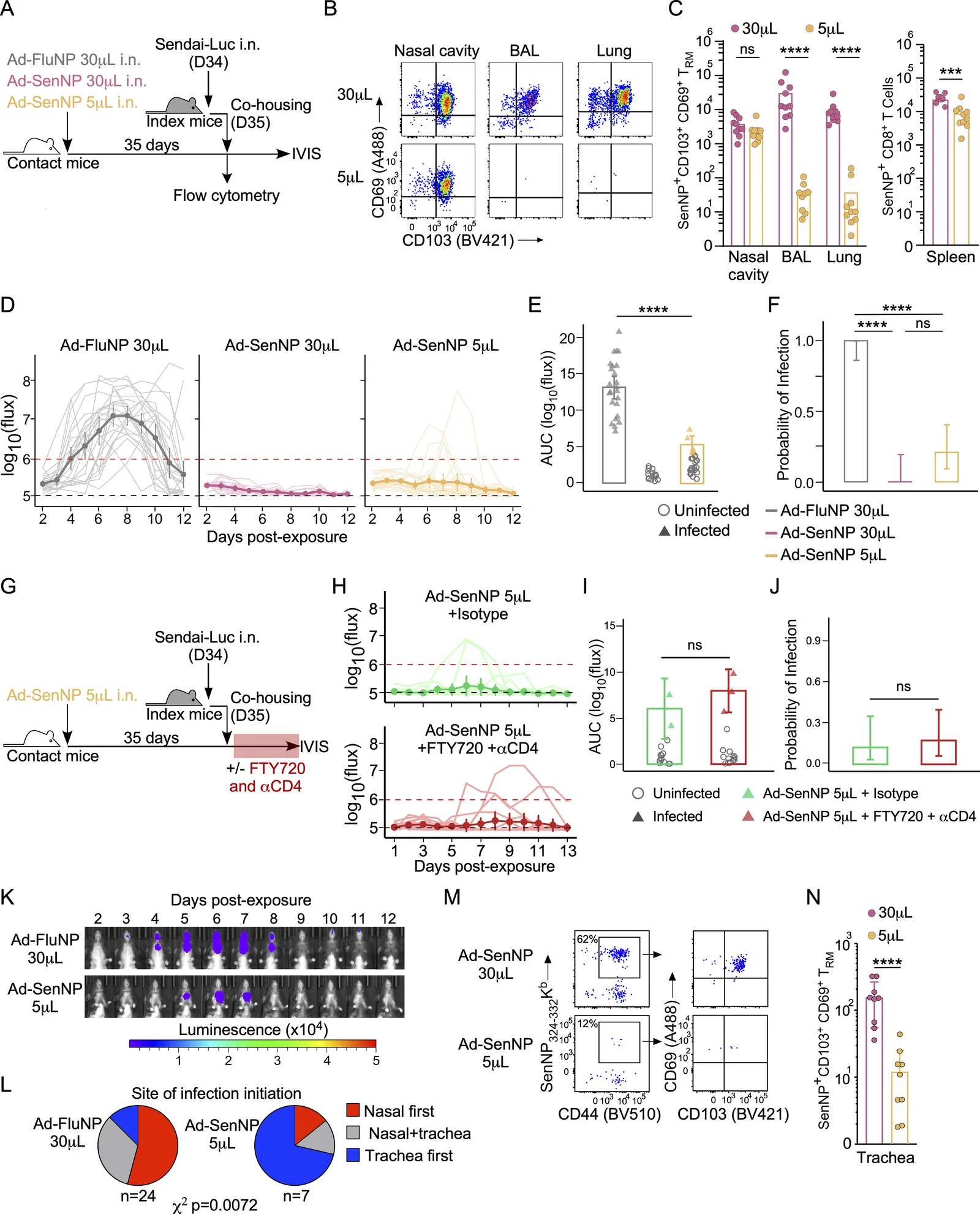
**CD8 TRM T cells in the URT alone are sufficient to prevent infection from transmission. (A)** Experimental schematic in which immunized contact mice were co-housed with a Sendai-Luc–infected index mouse. **(B)** Representative flow cytometry plots of CD103 and CD69 expression gated on IV^−^ CD8^+^ SenNP^+^ T cells in Ad-SenNP 30 and 5 μl immunized mice. **(C)** Number of CD103^+^ CD69^+^ SenNP^+^ TRM in the nasal cavity, BAL, lung, and splenic CD8^+^ SenNP^+^ cells of Ad-SenNP 30 μl (*n* = 10) and 5 μl (*n* = 10) immunized mice. **(D)** Bioluminescence curves of Ad-FluNP 30 μl (*n* = 24), Ad-SenNP 30 μl (*n* = 16), and Ad-SenNP 5 μl (*n* = 24) immunized contact mice when co-housed with Sendai-Luc–infected index mouse. **(E)** AUC of bioluminescence in immunized contact mice that became infected following co-housing. Grey circles represent uninfected mice, and triangles represent infected mice. **(F)** Probability of infection in the respiratory tract for immunized contact mice, calculated as the proportion of contact mice that become infected. **(G)** Experimental schematic in which contact mice were immunized with 5 μl Ad-SenNP and treated with FTY720 and anti-CD4 or vehicle and isotype controls. **(H)** Bioluminescence curves of Ad-SenNP 5 μl immunized contact mice treated with FTY720 and anti-CD4 (*n* = 16) or vehicle and isotype controls (*n* = 15) when co-housed with Sendai-Luc–infected index mouse. **(I)** AUC of bioluminescence in immunized contact mice that became infected following co-housing. **(J)** Probability of infection in the respiratory tract for immunized contact mice. **(K)** Representative bioluminescence images showing the location of Sendai-Luc replication in Ad-FluNP control mice or Ad-SenNP 5 μl immunized mice that had a breakthrough infection. **(L)** Proportion of infections that initiated in the nasal region, tracheal region, or nasal and tracheal regions concurrently in Ad-FluNP control (*n* = 24) and Ad-SenNP 5 μl immunized breakthrough (*n* = 7) mice. **(M and N)** Representative flow cytometry plots (M) and quantification (N) of SenNP^+^ CD69^+^ CD103^+^ CD8 T cells in the trachea of Ad-SenNP 30 μl and Ad-SenNP 5 μl immunized mice (*n* = 9 each) 35 days after immunization. For D and H, solid dark lines represent means, solid pale lines represent individual mice, dashed grey lines represent the limit of detection, and dashed red lines represent the threshold of infection. Error bars (E–I) represent 95% binomial confidence intervals. Data are combined from two (B and C and H–J), or three (D–F) independent replicates. Statistical significance was determined using a two-sided Mann–Whitney test (C, E, F, I, J, and N) or a chi-squared test (L), with ***P < 0.001, ****P < 0.0001, and NS for not significant.

**Figure S3.**
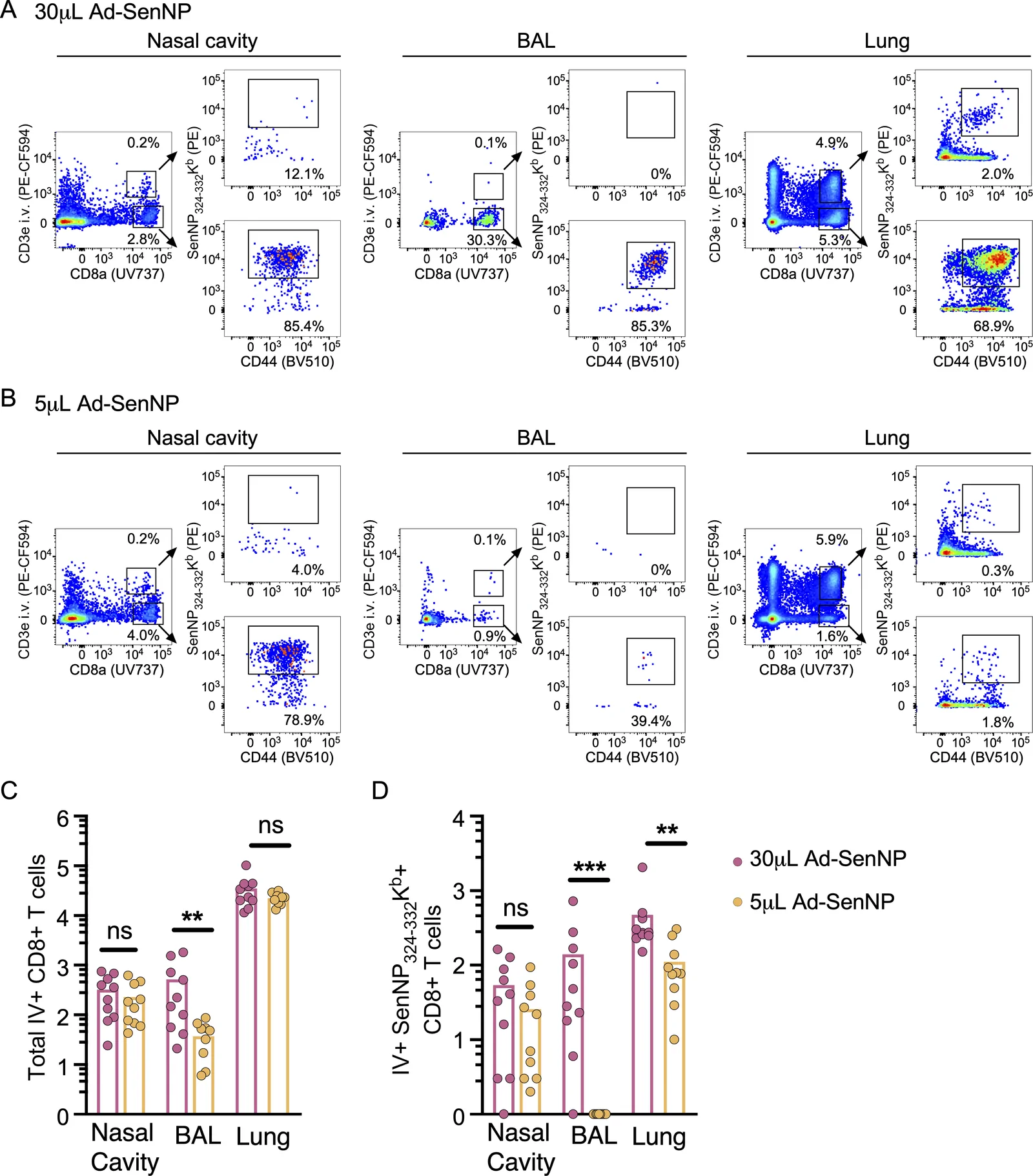
**IV^+^ CD8** ^**+**^ **T cells in the respiratory tract of Ad-SenNP 30 μl and 5 μl i.n. immunized mice. (A and B)** Representative flow cytometry plots showing the frequency of IV^+^ or IV^−^ SenNP^+^ CD8^+^ T cells in the nasal cavity, BAL, and lung of Ad-SenNP 30 μl (A) and Ad-SenNP 5 μl (B) immunized mice. **(C and D)** Quantification of total IV^+^ CD8 T cells (C) or IV^+^ SenNP^+^ CD8 T cells (D) in the nasal cavity, BAL, and lung of Ad-SenNP 30 μl (*n* = 10) and Ad-SenNP 5 μl (*n* = 10) immunized mice. Statistical significance was determined using a two-sided Mann–Whitney test with **P < 0.01, ***P < 0.001, and NS for not significant.

The observations that CD8 TRM protect against respiratory virus transmission and that antigen-specific CD8 T cell activation is restricted to the URT led us to ask whether URT TRM alone could prevent infection from transmission. The 30 μl i.n. inocula used throughout this study delivered antigen to the entire respiratory tract and elicited CD8 TRM in both the URT and LRT (total respiratory tract, TRT). To induce a similar number of Sendai virus-specific CD8 TRM in the URT without inducing an antigen-specific TRM response in the LRT, we used a low-volume 5 μl i.n. immunization with replication-deficient Ad-SenNP (Fig. 5, A and C). Comparable numbers of IV^+^ SenNP^+^ CD8^+^ T cells were generated in the URT for each condition (Fig. S3, A–D). Immunized mice were then co-housed with Sendai-Luc–infected index mice, and transmission outcomes were measured by in vivo imaging. As expected, 100% of animals (24 out of 24) that received the Ad-FluNP control became infected, and 0% of animals (16 out of 16) that received the 30 μl TRT immunization became infected. Animals with only URT TRM were largely protected against transmission, with only 20% (5 out of 24) becoming infected, an outcome which was not significantly different from that seen with TRT immunization (Fig. 5, D and F). Additionally, animals with only URT TRM that became infected exhibited significantly lower viral burdens than control mice (Fig. 5 E). To verify the ability of URT TRM to protect against transmitted virus in isolation, without circulating lymphocytes or CD4 T cells, we treated URT-only–immunized mice with FTY720 and CD4–depleting antibody (Fig. 5 G). CD4 T cell depletion throughout the respiratory tract and circulation was confirmed by flow cytometry (Fig. 2 C). In concordance with our previous results, protection against transmitted virus was evident in both groups. Infection was not detected in 86% (13 out of 15) of PBS and isotype control animals and 81% (13 out of 16) of FTY720- and CD4-depleted animals, and no difference was observed in the probability of infection (Fig. 5, H–J).

The in vivo bioluminescent images of the 7 URT TRM-only mice that became infected (*n* = 5 from Fig. 5 D and *n* = 2 from Fig. 5 H) revealed that infection bypassed the nasal region and remained isolated in the tracheal region in most mice (5 out of 7, 71%) (Fig. 5, K and L). Comparatively few infections in the Ad-FluNP control group initiated in the trachea (3 out of 24, 12%), while the majority initiated in the nasal region (13 out of 24, 54%) or nasal and tracheal regions concurrently (8 out of 24, 33%) (Fig. 5 L). To gain further insight into these results, we analyzed tracheal TRM in TRT- and URT-only–immunized mice. Surprisingly, TRT immunization generated a modest but discernible population of SenNP-specific tracheal TRM, while URT-only immunized mice had a scarcity of tracheal TRM (Fig. 5, M and N). Overall, these findings demonstrate that URT-resident memory CD8 T cells are sufficient to protect against respiratory virus transmission.

Previous studies have shown that i.n. T cell–mediated vaccines can abrogate pathology and reduce viral burdens of respiratory virus infections (Gilchuk et al., 2016; Luangrath et al., 2021; Wu et al., 2014). However, these studies often use high dose, i.n. challenge models that do not accurately mimic the dynamics of viral transmission and propagation in nature. Here, we use a respiratory virus transmission challenge model to demonstrate that CD8 TRM can act as sentinels of the respiratory tract to prevent the propagation of infection, independent of B cell responses, CD4 T cell help, or circulating immune cells. Understanding which respiratory tract tissues require immune surveillance to prevent infection is crucial to develop i.n. vaccines that stop the chain of transmission within populations. To this end, we show that URT CD8 TRM are preferentially activated during respiratory virus transmission and are sufficient to prevent infection. In accordance with an emerging body of literature on nasal-mediated immunity (Lim et al., 2025; Odle et al., 2024; Ramirez et al., 2024; Rha et al., 2024), our data support the development of URT T cell–based vaccines for respiratory viruses.

While URT CD8 TRM were largely effective in protecting against transmission, a handful of breakthrough infections, located in the trachea, occurred in these mice. Importantly, we noted that TRT immunization generated a minor population of tracheal TRM, while URT-only immunized mice lacked TRM-mediated immune surveillance in this tissue site. These findings provide additional insight into why trachea-localized breakthrough infections were observed in URT-only immunized mice, but infrequent in TRT-immunized mice. We propose that in the absence of T cell–mediated immune surveillance, the opportunity for trachea-localized infections to establish would be dependent on the mode of transmission. Airborne transmission may enable infectious respiratory particles to bypass nasal mucosal membranes and travel deeper within the respiratory tract, while direct deposition of nasal or oral secretions and contact transmission may result in viral particle seeding within the nasal epithelium (Gralton et al., 2011; Leung, 2021; Vargas-Maldonado et al., 2026). Our model does not differentiate between these modes of transmission, as index and contact mice freely interact with each other for the duration of the experiment, although previous work with Sendai virus has shown that both modes of transmission are highly efficient (Burke et al., 2013). We hypothesize that these breakthrough infections in the trachea of URT-immunized mice are a result of aerosol transmission alone. Evidence suggests that while aerosols play a role, the transmission of respiratory viruses in humans often involves contact with contaminated surfaces or deposition of large respiratory particles in the URT (Abney et al., 2025; Boone and Gerba, 2007). Thus, we theorize that URT CD8 TRM-mediated protection would impair the spread of circulating respiratory viruses.

The mouse nasal cavity is partitioned into posterior olfactory epithelium and anterior respiratory epithelium, with the latter containing the nasal-associated lymphoid tissue, nasal turbinates, septum, and maxillary sinus (Mettelman et al., 2022; Wellford et al., 2022). After influenza virus infection, CD103^+^ URT CD8 TRM reside in each of the aforementioned regions of the nasal tissue where they are poised to rapidly respond to challenge with their cognate antigen (Pizzolla et al., 2017a). It is possible that URT CD8 TRM localized around the conducting air passages of the nose play a more fundamental role in limiting respiratory virus transmission than those located deeper within the tissue, aligning with the earliest events during pathogen entry. A spatial understanding of URT CD8 TRM activation upon initial recognition of transmitted virus would inform vaccine design and correlates of protection for human vaccination. Human influenza challenge and transmission studies, where human participants are directly infected with influenza in controlled environments and the modes of transmission to exposed participants are analyzed, will prove highly useful in deciphering how different routes of viral transmission impact the localization of immune responses in the context of human infection (Killingley et al., 2012; Liebowitz et al., 2020; McIlwain et al., 2021; Nguyen-Van-Tam et al., 2020; Shetty et al., 2024). Recent studies in ferrets showed that transmissible influenza virus originates from the nasal cavity as opposed to the lung (Lakdawala et al., 2015; Richard et al., 2020; Xie et al., 2022). Based on these findings, we believe it is reasonable to assume that URT CD8 TRM would also limit transmission from infected individuals in addition to preventing infection upon exposure, as we have illustrated here.

To develop an i.n. URT-targeted vaccine, understanding the mechanisms of TRM differentiation and maintenance in the URT would inform vaccine formulation and adjuvant selection. In the lungs following i.n. adenoviral vector vaccination, alveolar macrophages are the predominant cellular population transduced to enable generation and maintenance of LRT CD8 TRM (Lobby et al., 2022). Previous studies have shown that URT TRM are primed in the cervical lymph nodes (Pizzolla et al., 2017b), but which APCs are involved, particularly for adenoviral vector vaccines, is unknown. FluMist, a commercially available LAIV vaccine delivered as a nasal spray, replicates more effectively at the colder temperatures found in the URT due to attenuating mutations (Fischer et al., 2015; Martinez-Sobrido et al., 2018). Although LAIV vaccination has been shown to induce nasal IgA responses in human vaccine recipients (Barría et al., 2013; Boyce et al., 1999; Thwaites et al., 2023), whether LAIV successfully induces URT CD8 TRM in humans is unknown, particularly in the context of pre-existing immunity. Additionally, an optimal i.n. vaccine should result in durable protection and long-lasting T cell responses. Although LRT CD8 TRM decline over time (Hayward et al., 2020; Slutter et al., 2017), our previous work demonstrates that URT CD8 TRM are more long-lived and can provide enduring protection against transmission in a mouse model (Uddbäck et al., 2024). One recent study was the first to provide evidence of URT CD8 TRM from nasal swabs of human SARS-CoV-2 vaccine recipients (Ramirez et al., 2024). Using such nasal swab sampling strategies, future studies should focus on understanding the longevity of URT CD8 TRM in humans to assess whether URT T cell–mediated vaccines would necessitate boosting regimens.

In our transmission model, vaccine-induced T cell responses are directed against a single SenNP epitope expressed by an LAIV or adenoviral vector. While this creates an ideal, reductionist model to study the roles of TRM in transmission, it does not encompass the entirety of the immune response to transmitted virus, which may include memory T cell and B cell responses to multiple viral epitopes, neutralizing antibodies, and antiviral innate responses (Tang et al., 2026; Zhang et al., 2026). The interplay and relative importance of each of these immune components for protection against viral transmission is not fully understood, and future studies utilizing systems immunology approaches or more complete immune landscapes are necessary to determine how these components interact and potentially compensate for each other to promote optimal immunity.

In summary, we have uncovered a novel role for nasal, URT CD8 TRM in preventing infection in a transmission model. We show that URT CD8 TRM are the predominant virus-specific T cell population responding to respiratory virus transmission and that, when present in sufficient quantities, can inhibit the establishment of infection. These findings have significant implications for the development of i.n. vaccines to prevent the spread of respiratory viruses.

## Materials and methods

### Mice

Female C57/BL/6J, B6.129S2-*Ighm*^*tm1Cgn*^/J (μMT^−/−^), and C57BL/6-Tg(Nr4a1-EGFP/cre)820Khog/J (Nur77^GFP^) mice were purchased from Jackson Laboratory and bred in-house. Mice were housed at Emory University under specific pathogen-free conditions and used in experiments between 8 and 12 wk of age. All experiments were conducted in accordance with the Institutional Animal Care and Use Committee guidelines of Emory University (PROTO201700581).

### Viruses

All i.n. infections were administered under anesthesia with isoflurane (Patterson Veterinary). Mice received 50,000 PFU of live-attenuated influenza A/Puerto Rico/8/34 virus (LAIV WT), 20,000 PFU of live attenuated influenza A/Puerto Rico/8/34 virus expressing SenNP FAPGNYPAL epitope (LAIV-SenNP), 50 PFU of influenza A/Puerto Rico/8/34 virus expressing SenNP FAPGNYPAL epitope (PR8-SenNP), 2 × 10^7^ PFU of replication-deficient adenovirus serotype 5 expressing the influenza nucleoprotein ASNENMETM epitope (Ad-FluNP), or 2 × 10^7^ PFU of replication-deficient adenovirus serotype 5 expressing the SenNP FAPGNYPAL epitope (Ad-SenNP) i.n. in a 30 μl volume, as previously described (Uddbäck et al., 2024). URT-only infections were performed using a 5 μl i.n. immunization of 2 × 10^7^ PFU of Ad-SenNP under isoflurane anesthesia. Index mice used in transmission experiments were infected i.n. with 1,500 PFU of Sendai virus encoding a luciferase reporter (Sendai-Luc) (Burke et al., 2011; Burke et al., 2013) in a 30 μl inoculum under isoflurane anesthesia.

### Chemical treatments and antibody depletion

For FTY720 treatments, mice were injected daily i.p. with 150 μg (Cayman Chemical) suspended in PBS (Dunbar et al., 2020), starting 3 days before initiation of co-housing. For T cell depletions, mice were given an initial loading dose of anti-CD4 mAb clone GK1.5 (BioXcell) or anti-CD8 mAb clone 2.43 (BioXcell) 200 μg i.n. and 200 μg i.p., followed by 200 μg i.p. every 3 days. Control groups were administered an isotype control antibody (BioXcell) at the same dose and schedule. 1 mg of EdU (Cayman Chemical) was administered 1 day prior to sacrifice i.p. in 200 μl PBS (Lobby et al., 2024).

### In vivo imaging

In vivo imaging was performed using an In Vivo Imaging System (IVIS) Lumina LT Series III (PerkinElmer) as previously described (Uddbäck et al., 2024). Mice were injected i.p. with 3 mg of IVISbrite D-Luciferin (Revvity) reconstituted in D-PBS, 10 min prior to image acquisition. Images were captured using an XFOV-24 lens, and acquisition settings were a binning of 8, F/stop of 1, with exposure times of 5, 30, and 120 s. Background bioluminescence was calculated by imaging 2 uninfected mice each day of the experiment. At least 1 day prior to co-housing for transmission experiments, chest hair was removed from mice by shaving and applying a depilation cream. Images were analyzed in the Living Image 4.7.2 software (Perkin Elmer), where region of interest gates were manually drawn around the respiratory tract region and used to quantitate bioluminescent flux. Data were exported to Microsoft Excel. Bioluminescent curves and statistics were performed using GraphPad Prism or R software.

### Plaque assays

Samples for nasal shedding analyses were collected by dipping the nose of isoflurane-anesthetized mice into a 12-well plate with 1% BSA in PBS. Samples were then frozen at −80°C until performing the assay. 12-well plates were seeded with 4 × 10^5^ Vero cells and grown to near 100% confluency. Cells were incubated with samples for 1 h at 34°C and 5% CO_2_, and then treated with a 1 x MEM, Hepes buffer, 0.5% gelatin, 0.5% agarose, 0.4% BSA, 1x GlutaMAX, 0.02 mg/ml penicillin-streptomycin, 0.3% sodium bicarbonate, and 5 U/ml TPCK trypsin overlay. After 4 days, the overlay was removed, cells were fixed with 10% formaldehyde and stained with 1% crystal violet. All samples were titrated in duplicate. Plaques were counted manually, and PFU was calculated by multiplying the average number of plaques by the dilution factor. The limit of detection was established as five plaques for the lowest dilution, equating to 25 PFU.

### Tissue collection, cellular isolation, and flow cytometry

Five minutes prior to euthanasia, mice were injected i.v. with 1.5 μg of anti-CD3ε-PECF594 clone 145-2C11 (BioLegend) or 2 μg CD45.2-BV650 clone 104 in 200 μl of PBS to distinguish circulating cells from tissue-resident cells. Mice were euthanized with a lethal i.p. injection of 2,2,2-tribromoethanol (Avertin) followed by brachial exsanguination. Lungs, BAL, nasal cavity, trachea, and/or spleen were harvested from each animal. Lungs and nasal cavities were enzymatically digested with Collagenase D (5 g/l; Roche) and DNase (1 × 10^6^ U/l; Sigma-Aldrich) for 30 min at 37°C. Trachea was enzymatically digested with Collagenase D (5 g/l; Roche), DNase (1 × 10^6^ U/l; Sigma-Aldrich), and Dispase (15u/ml; Thermo Fisher Scientific) for 30 min at 37°C. Lymphocytes were further isolated from lungs using 40%/80% Percoll density centrifugation. Cells were filtered through a 70-μm membrane, and RBCs were lysed using ACK buffer prior to staining. For flow cytometry and FACS, cells were F_c_ blocked using murine anti-CD16/32 2.4.G2 for 10 min. Samples were then stained with 1:100 Sendai-NP (K^b^_324-332_) tetramer- PE or APC (provided by NIH Tetramer Core Facility) for 1 h. Extracellular staining with 1:100 dilutions of fluorescently conjugated antibodies including anti-CD8α-BV711 clone 53–6.7, CD4-UV496 clone GK1.5, CD4-A700 clone RM4-4, CD103-BV421 clone 2E7, CD69-A488/PE-Cy7 clone H1.2F3, CD62L-BV605 clone MEL-14, CD44-BV510 clone IM7, CD19-APC eFluor780 clone 1D3, CD3-APC clone 17A2, and CD45-A700/PerCPCy5.5 clone 30-F11 was performed for 30 min. Zombie NIR or 7-aminoactinomycin D was used to determine cellular viability. For EdU experiments, samples were further stained using the Click-iT Plus EdU Flow Cytometry Kit – Alexa Fluor 647 (Invitrogen) according to the kit’s standard protocol. Cell counts were calculated manually using a hemocytometer for nasal cavity, BAL, and trachea samples or a LUNA-II automatic cell counter (Logos Biosystems) for spleen and lung tissues. All samples were acquired on a Fortessa X20 flow cytometer or sorted on a FACSAriaII (BD Biosciences). SenNP^+^ TRM were gated on singlets, lymphocytes, i.v. label-live cells, CD4^−^CD8α^+^, CD44^hi^ Tetramer^+^, and CD69^+^ CD103^+^. Flow cytometry data were analyzed using FlowJo version 10 software.

### Single-cell RNA sequencing

Single-cell RNA-sequencing gene expression libraries were generated following the Chromium Single Cell 5′ Reagent Kits User Guide, version 2 Chemistry Dual Index (10x Genomics). Illumina fastq files were processed and mapped to the GRCh38-2024-A reference transcriptome using 10x Genomics Cell Ranger 8.0.0 (Zheng et al., 2017), and each biological replicate was aggregated on a per-batch basis for downstream analysis using the “aggr” command in Cell Ranger with the default parameters. Aggregated data were analyzed using Seurat version 5.0.0 (Hao et al., 2024). Subsequent command references are from this software, and default parameters were used for all commands unless otherwise specified. Low-quality cells with the following criteria were filtered out: percent of mitochondrial reads in each cell >5% and <500 (replicate 1) and 300 (replicate 2) uniquely expressed genes per cell. The remaining counts from each batch were separately normalized and scaled, and the batch-corrected, integrated UMAP was created using Harmony (Korsunsky et al., 2019) and clustered using the top four principal components of the dimensional reduction with a resolution of 0.24 that resulted in six clusters. RNA velocity analysis was performed by first estimating the quantity of unspliced and spliced reads on a per-sample basis with velocyto version 0.17.15 (La Manno et al., 2018). Then, using scvelo version 0.2.5 (Bergen et al., 2020), RNA velocity was computed with a steady-state model. The vector field of the RNA velocity was averaged into streamlines and overlaid on the 2D UMAP for visualization of broader flows, and additional metadata information about the cells was underlaid. All sequencing data are available from the National Center for Biotechnology Information Gene Expression Omnibus (GEO) under accession no. GSE329262.

### Statistical analyses

Statistical analyses were performed using GraphPad Prism version 9 or R software. For in vivo imaging analyses, the threshold of infection was calculated as the mean background bioluminescence flux +2.5 × standard deviation of the background flux. Any flux values below the mean background flux were replaced by the mean background flux. Area under the curve (AUC) for each animal was calculated using the trapz function in R, and the background AUC was subtracted. AUC values were compared using a Mann–Whitney test with the wilcox_test function in R. The probability of infection was calculated as the proportion of mice in each group that became infected and had bioluminescent flux values greater than the threshold of infection. Confidence intervals were generated using a binomial probability distribution with the binconf function in R. Probability of infection values were compared using a proportion test with the prop_test function in R. Unless otherwise indicated, data points represent individual mice for at least two independent replicates. Statistical significance for cell number analyses was determined using a two-sided Mann–Whitney test in GraphPad Prism. P values for all statistics are indicated as *P < 0.05; **P < 0.01; ***P < 0.001; and ****P < 0.0001; N.S., not significant.

### Online supplemental material

Fig. S1 shows that protection against transmission occurs independently of circulating effector cells, B cells, and CD4 T cells even when a lower quantity of respiratory tract TRM are present following LAIV-SenNP i.n. immunization, in support of Fig. 2. Fig. S2 contains the flow cytometry gating strategy for nasal IV^−^ CD8^+^ SenNP^+^ CD69^+^ CD103^+^ TRM and validation of FTY720 and anti-CD4 depletion efficacy in the nasal cavity and lung, in support of Figs. 2 and 5. Fig. S3 illustrates CD8^+^ T cells in the IV^+^ compartment of the nasal cavity, BAL, and lung of Ad-SenNP 30 μl and Ad-SenNP 5 μl i.n. immunized mice, in support of Fig. 5.

## Data Availability

Single-cell RNA sequencing data are available from the NCBI GEO under accession no. GSE329262 (Fig. 4). All other data are available in the published article and the online supplementary materials.
